# Metal-Organic Frameworks as Versatile Heterogeneous Solid Catalysts for Henry Reactions

**DOI:** 10.3390/molecules26051445

**Published:** 2021-03-07

**Authors:** Francisco G. Cirujano, Rafael Luque, Amarajothi Dhakshinamoorthy

**Affiliations:** 1 Institute of Molecular Science (ICMOL), Universidad de Valencia, 46980 Paterna, Valencia, Spain; 2 Departamento de Quimica Organica, Universidad de Cordoba, E-14014 Cordoba, Spain; q62alsor@uco.es; 3 School of Chemistry, Madurai Kamaraj University, Madurai, Tamil Nadu 625 021, India

**Keywords:** metal–organic frameworks, Heterogeneous catalysis, Henry reaction

## Abstract

Metal–organic frameworks (MOFs) have become one of the versatile solid materials used for a wide range of applications, such as gas storage, gas separation, proton conductivity, sensors and catalysis. Among these fields, one of the more well-studied areas is the use of MOFs as heterogeneous catalysts for a broad range of organic reactions. In the present review, the employment of MOFs as solid catalysts for the Henry reaction is discussed, and the available literature data from the last decade are grouped. The review is organized with a brief introduction of the importance of Henry reactions and structural properties of MOFs that are suitable for catalysis. The second part of the review discusses the use of MOFs as solid catalysts for the Henry reaction involving metal nodes as active sites, while the third section provides data utilizing basic sites (primary amine, secondary amine, amides and urea-donating sites). While commenting on the catalytic results in these two sections, the advantage of MOFs over other solid catalysts is compared in terms of activity by providing turnover number (TON) values and the structural stability of MOFs during the course of the reaction. The final section provides our views on further directions in this field.

## 1. Introduction

**Scheme 1:**
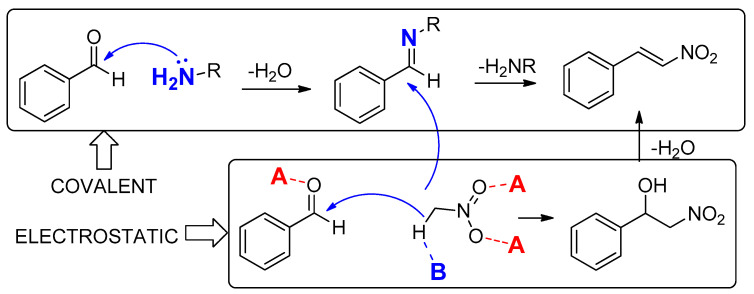
Nucleophilic RNH_2_ amines (covalent catalysis) or acid (A)/base (B) catalysts (electrostatic catalysis) promote the nitroaldol condensation (Henry reaction).

Among the synthetic methodologies available for creating C-C bonds in organic molecules, acid-base catalysed aldol condensations are traditionally the most widely employed. In particular, nitroaldol condensation (known as the Henry reaction) represents a powerful method for the incorporation of N-containing groups into the target molecule (i.e., with the nitro group being a potential precursor to the ubiquitous amino group through a simple reduction step). The Henry reaction consists of the nitroaldol condensation between a nitroalkane, with a α-H to the nitro group, and a carbonyl group, usually an aldehyde (Scheme 1). The nitroalcohol formed during the C-C coupling usually dehydrates into the nitroalkene, depending on the catalyst and reaction conditions (basicity, solvent, temperature, etc.). This reaction originally employed stoichiometric amounts of inorganic bases, such as hydroxides or hydrogen carbonates [1], which present important drawbacks associated with: (i) the formation of stoichiometric amounts of salts, (ii) the impossibility of recovering and recycling the base, and (iii) the tedious work-up with large amounts of organic solvents in order to isolate the product. Therefore, the use of a catalyst in low concentrations with respect to the reaction substrates is desired in order to increase the productivity or TON of this reaction, i.e., molecules of nitroalkene produced per active site. Solid catalysts are preferred over soluble ones, since the recovery and recycle of heterogeneous catalysts is simpler and offers the possibility of working with a continuous flow and under broader and safer operational conditions. In general, two pathways are usually employed to catalyze the Henry reaction: (i) the formation of imine intermediates by reaction of the carbonyl group with catalytic amine sites (covalent pathway in Scheme 1); (ii) electrostatic interactions between acid and base sites with the aldehyde and nitroalkane (electrostatic pathway in Scheme 1). While the first pathway only applies to amine catalyst, the second one refers to Brønsted/Lewis acid-base catalysts, as well as hydrogen bond catalysts.

Metal–organic frameworks (MOFs) are porous and crystalline solids with a high versatility in terms of composition and structure. Their high surface area and possibility of introducing different types of well-defined active sites (e.g., metals, organic functions or organometallic complexes) resulted in the exponential growth of their catalytic applications over traditional oxides, carbon or polymers [2,3,4,5]. In particular, the formation of a C–C bonds in the manufacture of high-added-value molecules using MOFs as heterogeneous catalysts is an important research field with applications in the fine chemical and pharmaceutical industries [6,7,8,9]. Besides the use of MOFs in gas storage [10], gas separation [11], sensors [12], proton conductivity [13], and chromatographic applications [14], heterogeneous catalysis is one of the areas that have been explored extensively in the last two decades. The types of reactions that have been reported using MOFs as heterogeneous catalysts include oxidation [15,16,17], reduction [18], condensation [19], C–C cross-coupling [20], C–O bond formation [21], C–N bond formation [21], esterification [22], acetalization [23,24], Friedel Crafts alkylation [25], CO_2_ fixation to fine chemicals [26,27] and photocatalysis [28,29]

Due to the versatile tailoring properties of MOFs and in continuation of the abovementioned reactions, MOFs have also been used as heterogeneous solid catalysts for Henry reactions. In particular, the presence of well-defined N- or O-containing groups, open metal sites, and hydrogen-bond-donating protic polar groups precisely attached to the framework has shown an excellent catalytic performance in the Henry reaction. In this review, we aim to summarize most of the work carried out in the last decade using MOFs as heterogeneous catalysts for the activation of carbonyl and nitro groups, and its further C–C coupling through the Henry reaction. Particular attention has been paid to the productivity of the different active sites present in MOFs, quantitatively comparing their performance based on the TON values under different reaction conditions. In addition, catalyst stability after the catalytic reaction was also discussed to forecast the benefits of employing MOFs as a solid catalyst compared to other related materials. Furthermore, the proof of stability after catalysis was also discussed with appropriate characteristic evidence.

In recent years, the activity of the MOF catalysts is often verified using Henry reaction as a model reaction to ascertain the presence of various functional groups. This reaction is catalyzed by different active sites, including: (i) metal nodes such as Lewis acids, (ii) basic functional groups (amino/amido) and (iii) synergistic involvement of the above two active sites. Hence, this review is divided based on the active sites responsible for promoting this reaction. The first part describes the use of MOFs as solid Lewis acid catalysts and the existing literature is organized based on the nature of metal nodes. Later, the second part provides the use of basic MOFs as solid catalysts and the available data are organized depending on their basic nature, such as primary amines, secondary amines, amides and urea sites. The final section summarizes the current state-of-the-art in this field and also provides our views for further development in this area.

## 2. Metal Nodes as Active Sites

### 2.1. Co Metal Nodes as Active Sites

**Scheme 2:**
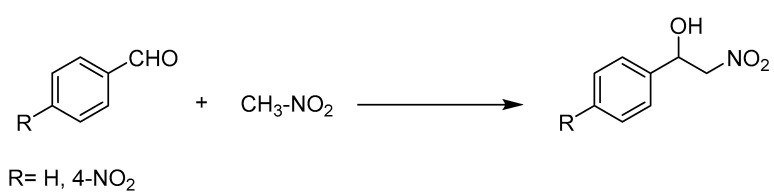
Henry reaction between (4-nitro) benzaldehyde and nitromethane.

A cobalt-based MOF Co_2_(bdda)_1.5_(OAc)_1_·5H_2_O (UoB-3) (bdda: 4,4′-[benzene-1,4-diylbis(methylidenenitrilo)] dibenzoic acid) was reported as a heterogeneous solid catalyst for the Henry reaction between benzaldehyde and nitromethane (Scheme 2) affording 83% yield in water at 70 °C after 24 h [30]. Furthermore, the catalytic performance was also tested between substituted aromatic aldehydes and nitromethane to obtain the respective β-nitroalkanols in 62 to 88% yields. Interestingly, the size-selective catalytic performance of UoB-3 was studied for the reaction of benzaldehyde with nitromethane (2.0 Å × 3.3 Å), nitroethane (2.2 Å × 3.9 Å) and nitropropane (2.2 Å × 5.6 Å). The catalytic data clearly indicated that the yield is decreased upon an increase in the molecular size of nitroalkanes. Thus, these results prove that the catalytic reaction occurs within the pores of the solid catalyst. The solid was used for five cycles with no significant drop in its activity. The FT-IR spectra of the fresh and reused solids remained identical. However, further characterizations should have provided a deeper understanding of catalyst stability. The reaction was heterogeneous in nature, as revealed by hot-filtration test.

### 2.2. Cd Metal Nodes as Active Sites

**Scheme 3:**
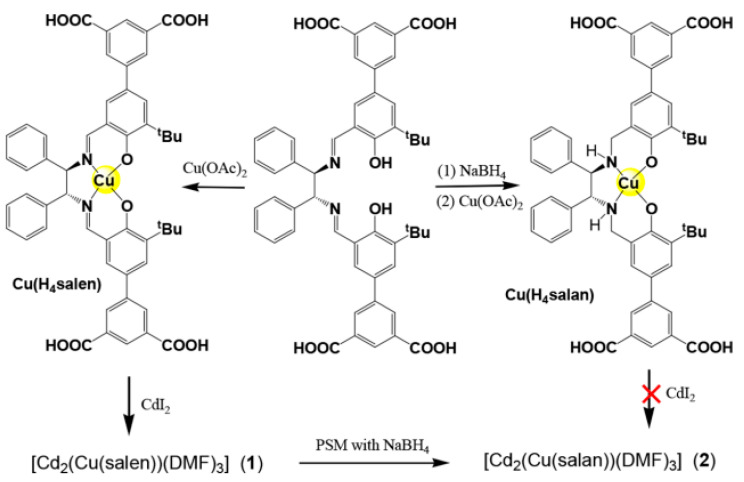
Synthesis of [Cd_2_(Cu(salen))(DMF)_3_]·DMF·3H_2_O and its post-synthetic reduction with NaBH_4_. Reproduced with permission from [31]. Copyright (2018) American Chemical Society.

A 3D chiral metallosalen-based MOF, [Cd_2_(Cu(salen))(DMF)_3_]·DMF·3H_2_O possessing a 1D open channel was prepared, and it was further post-synthetically reduced with NaBH_4_ to selectively convert imine to amine without affecting the porous framework (Scheme 3) [31]. Among the various reaction conditions screened for the Henry reaction between benzaldehyde and nitromethane at 45 °C in methanol, 70% yield of the desired product with 95% ee was achieved after 48 h. In contrast, the activity of [Cd_2_(Cu(salen))(DMF)_3_]·DMF·3H_2_O (without reduction) was relatively lower, at 47% yield with 87% ee value under identical conditions. Furthermore, the post-synthetically modified solid was used as a heterogeneous solid catalyst for the asymmetric Henry reaction with broad range of aldehydes with high yields (mostly >90%) and ee values (>90%) compared to the unreduced solid under similar conditions. Interestingly, the reaction between 9-anthraldehyde and nitromethane provided a significantly lower yield (11%) with 45% ee value due to the diffusion limitation of this bulky aldehyde, thus indicating size-dependent catalysis. Hot filtration experiment indicated the heterogeneity of the reaction. The solid was recycled at least five times with no notable decay in its activity and ee values. Furthermore, the powder XRD pattern of the recovered solid was identical to the fresh solid, and, hence, the structural integrity of the catalyst during the reaction was retained.

**Scheme 4:**
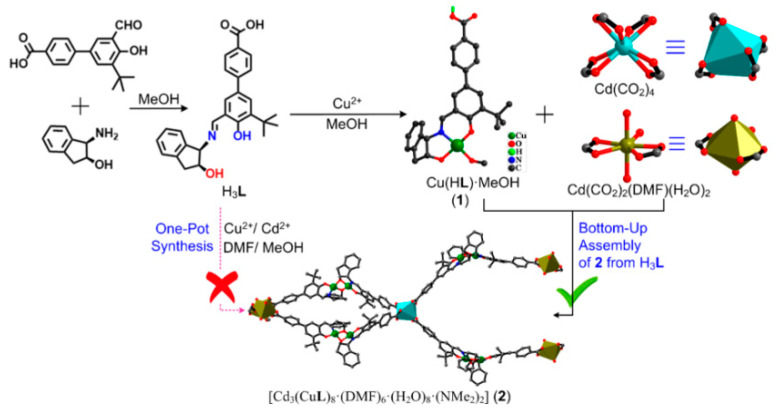
Synthesis of tridentate Schiff base ligand H3L, mononuclear copper complex **1** and MOF **2**. Reprinted with permission from [32]. Copyright (2018) American Chemical Society.

Recently, a bottom-up assembly of a monomeric copper complex with a 2D heterometallic MOF from a carboxylate-functionalized tridentate Schiff base ligand and metal ions was developed, and the resulting 2D MOF was found to be an efficient heterogeneous catalyst for Henry reaction [32]. (Scheme 4) Benzaldehyde and its derivatives with electron-withdrawing groups facilely reacted with nitromethane, in the presence of this 2D MOF, to their respective β-nitroalcohols in 86–97% yields in methanol after 12 h. In contrast, the activity of the mononuclear complex (with an identical loading in 2D MOF) was 48 and 53% yields for the reaction between benzaldehyde and 4-nitrobenzaldehyde with nitromethane, respectively. The superior activity of this solid compared to its homogeneous counterparts for different aldehydes is due to the dual activation of the substrates by the active bimetallic copper center confined in the MOF network. It was believed that the poor activity of the homogeneous complex was due to the low probability of the collision of activated nucleophile and electrophile.

Homochiral MOFs based on Cd(II), [{Cd_2_(Lglu)_2_(bpe)_3_(H_2_O)}·2H_2_O] and [{Cd_3_(L-glu)_2_(bpe)_3_(H_2_O)}.2NO_3_.H_2_O] (L-glu: L-glutamate dianion; bpe: 1,2-bis(4-pyridyl)ethylene) were prepared under solvothermal conditions [33]. The activity of [{Cd_2_(Lglu)_2_(bpe)_3_(H_2_O)}·2H_2_O] was assessed in the Henry reaction of benzaldehyde with nitroethane using 10 mol% of Zn(II) salt as a co-catalyst to obtain the corresponding β-nitroalcohol in 89.3% yield after 72 h in methanol at room temperature. The control experiments indicated that the addition of Zn(II) salt is essential, since Cd(II) nodes in [{Cd_2_(Lglu)_2_(bpe)_3_(H_2_O)}·2H_2_O] are completely connected and do not offer accessibility to the reactants. The solid also exhibited a wide substrate scope, with the conversions ranging between 67 and 100%. The reaction was found to be heterogeneous, and the catalyst retained its activity in four consecutive cycles. Powder XRD of the recycled solid matched with the fresh solid, thus suggesting the retainment of structural integrity during the reaction.

### 2.3. Zn Metal Nodes as Active Sites

The reaction of Zn(NO_3_)_2_ with the tripodal ligand, 1,3,5-tri(4-carboxyphenoxy)benzene) (TCPB) resulted in two porous isoreticular MOFs, [Zn_3_(TCPB)_2_·2DEF]·3DEF and [Zn_3_(TCPB)_2_·2H_2_O]·2H_2_O·4DMF. The structural analysis of these solids has shown that coordinatively unsaturated metal sites can be generated upon the removal of adsorbed solvent molecules, thus they can behave as Lewis acids [34]. The catalytic activity of desolvated [Zn_3_(TCPB)_2_·2H_2_O]·2H_2_O·4DMF was tested between 4-nitrobenzaldehyde with nitroalkanes with different molecular dimensions. The experimental results have shown that the conversion is 40, 15 and 3% for the reaction of 4-nitrobenzaldehyde with nitromethane, nitroethane and nitropropane, respectively, at 70 °C after 72 h. No efforts were made to probe the catalytic activity of this solid with different aldehydes, and catalyst stability was also not reported.

Two zinc(II)-based fluorinated MOFs, namely, [Zn_2_(hfipbb)_2_(4-bpdh)].(DMF)0.5 (TMU-55) and [Zn_2_(hfipbb)_2_(4-bpdb)].(DMF)_2_ (HTMU-55) with micro- and nanorod morphology were synthesized using ultrasonic waves at room temperature [35]. The catalytic activity of these MOFs was studied in the Henry reaction between benzaldehyde and nitromethane. TMU-55 and HTMU-55 exhibited 71% and 68% conversions, respectively, in methanol at 60 °C after 24 h. The activity of these two solids is due to the basicity from azine groups and their better interaction with substrate. In contrast, nanostructures of TMU-55 and HTMU-55 displayed 97 and 87% conversions, respectively, under identical conditions, and this enhanced activity is due to the higher surface area. Both solids in bulk and nanostructures were reusable at least three times without any decay in their activity. Powder XRD patterns and SEM images of the recovered catalyst were similar to the fresh catalyst, thus suggesting the maintenance of structural integrity and morphology.

### 2.4. Cu Metal Nodes as Active Sites

In one of the earlier studies, the reaction of mixed-linkers, namely, pyridine-2,3,5,6-tetracarboxylic acid (pdtc) and (E)-4-(2-(pyridin-4-yl)vinyl)benzoic acid (L), with copper atoms afforded a porous 3D MOF structure [Cu_3_(pdtc)L_2_(H_2_O)_3_].2DMF.10H_2_O [36]. The structural analysis of this solid clearly indicated that the copper coordination sites are occupied by volatile water molecules, which can be evacuated upon thermal treatment to generate Lewis acid sites. Hence, the catalytic activity of desolvated [Cu_3_(pdtc)L_2_(H_2_O)_3_].2DMF.10H_2_O solid was examined for the reaction of 4-nitrobenzaldehyde with nitromethane and 85% yield was observed under solvent-free conditions at 70 °C after 36 h. Further, this solid was also able to promote the Henry reaction with various benzaldehydes and nitroalkanes under similar conditions. Heterogeneity of the reaction was proved by hot filtration test. The solid was repeatedly used for up to five cycles with no notable decrease in its yield. Powder XRD of the recovered solid remained identical to the fresh catalyst; thus, the structural integrity is retained.

In another precedent, the molecular-sieving effect of HKUST-1 was reported in the Henry reaction between aromatic aldehydes with nitromethane. The experimental results have shown that the reaction of salicylaldehyde with nitromethane, in the presence of HKUST-1, to obtain trans-nitrovinylphenol, is much faster, while no reaction occurred with 9-anthracenecarbaldehyde [37]. The difference in catalytic performance of HKUST-1 with salicylaldehyde and 9-anthracenecarbaldehyde is due to the sieving effect of this catalyst, which clearly discriminates by size. This is an interesting example, illustrating that the reaction does not occur exclusively with exterior catalytic sites.

A 3D NbO-type porous Cu-MOF possessing tertiary amine groups and paddle wheel dinuclear Cu_2_(COO)_4_ secondary building units was prepared, and its activity was reported in the Henry reaction for the synthesis of β−nitroalcohols [38]. A wide range of aldehydes was reacted with nitromethane in the presence of the activated Cu-MOF to afford the corresponding β−nitroalcohols under solvent-free conditions at 50 °C after 48 h. The highest yield of 89% was achieved for the reaction between 4-nitrobenzaldehyde and nitromethane under similar conditions. A series of control experiments proved that the higher activity of this solid is derived from the synergistic effect of acidic and basic sites. The solid catalyst was reused for at least four cycles. Although the crystallinity was retained for up to three cycles, a minor degradation was seen after the fourth cycle, as evidenced by powder XRD. On the other hand, hot filtration test indicated he heterogeneity of the reaction using this solid catalyst.

**Figure 1:**
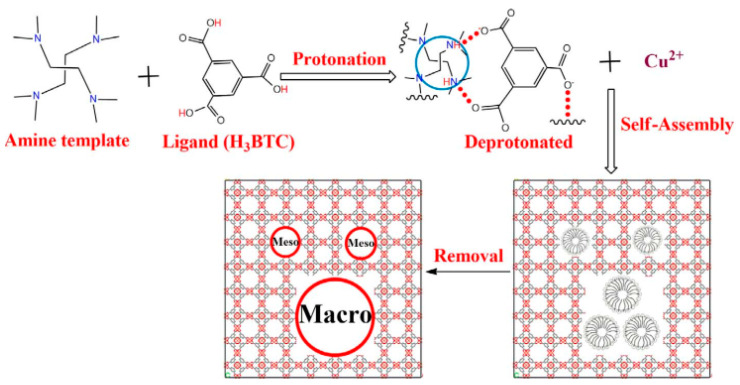
Possible mechanism for the synthesis of hierarchical porous MOFs using amine templates. Reprinted with permission from [39] Copyright (2018) Elsevier.

**Figure 2:**
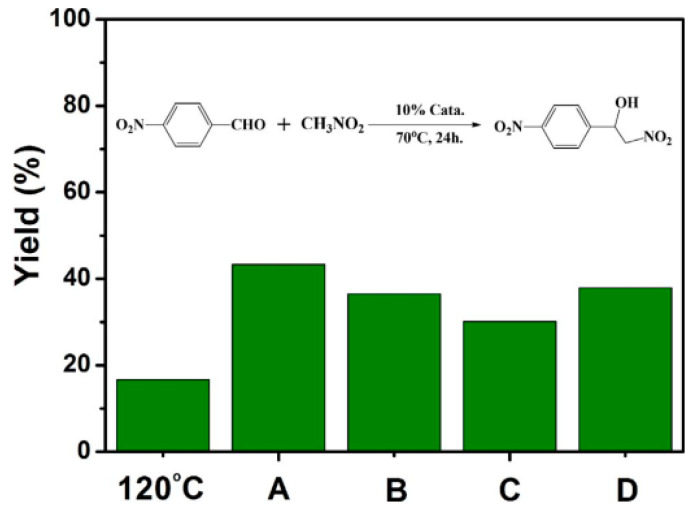
Twenty-four-hour yield of the Henry reaction of 4-nitrobenzaldehyde with nitromethane catalyzed by Cu-BTC_X: 120 °C corresponds to conventional microporous Cu-BTC; A (Cu-BTC_A); B (Cu-BTC_B); C (Cu-BTC_C); D (Cu-BTC_D) in which A, B, C and represents aniline, 2- dimethylaminoethylamine, hexylamine and N,N,N′,N′-tetramethylethylenediamine as templates for the preparation of hierarchical MOFs. Reprinted with permission from [39]. Copyright (2018) Elsevier.

A simple and green protocol was developed for the synthesis of hierarchical porous MOFs of Cu-BTC with tunable morphologies and porosities including meso- and macropores and properties. Among the various organic amines used for the preparation of this hierarchical porous Cu-BTC, the use of aniline resulted in better activity. Furthermore, due to the presence of micro-, meso-, macropore and high thermal stability, hierarchical Cu-BTC showed more than two-fold activity compared to conventional Cu-BTC using exclusively micropores in a Henry reaction between 4-nitrobenzaldehyde and nitromethane (Figure 1) [39]. Interestingly, the reaction between 4-bromobenzaldehyde and nitromethane using hierarchical porous Cu-BTC_A afforded two-fold higher performance than for microporous Cu-BTC_120 °C MOF (Figure 2). These results clearly indicate the benefits of hierarchical MOFs with a large number of meso- and macropores to facilitate the diffusion of bulky reactants or products. The Cu-BTC_D solid also retained its activity for five cycles.

Setting another precedent, the same research group prepared a stable, hierarchically porous Cu-BTC using the anionic surfactant sodium dodecylbenzene sulfonate as a template with the generation of meso- and macropores [40]. Interestingly, the mesoporosity of HP-MOFs was tuned from 7 to 24 nm by adjusting the amount of template. The catalytic activity of hierarchical Cu-BTC was 1.5-fold higher than the conventional Cu-BTC for the Henry reaction between 4-nitrobenzaldehyde with nitromethane in water medium.

HKUST-1@MMS (MMS: mesoporous silica matrix) hybrid material was prepared by embedding HKUST-1 nanoparticles within the channel of mesoporous silica. MMS matrix was employed as a structure-directing agent, which ensures the formation of small HKUST-1 nanoparticles in the range of 4–8 nm with uniform distribution inside silica mesopores. The activity of HKUST-1@MMS was examined in the Henry reaction between 4-nitrobenzaldehyde and nitromethane, observing 91% yield in water at 75 °C after 48 h [41]. The activity of HKUST-1@MMS, HKUST-1 and MMS was 44, 1 and 3% yields, respectively, with nitromethane as a solvent, at 50 °C after 48 h. The superior activity of HKUST-1@MMS hybrid catalyst was due to the synergistic effect between Lewis acid sites of Cu^2+^ ions in HKUST-1 and weak Brønsted acidic sites of MMS. No significant changes in the XRD pattern for the three times using a hybrid solid was observed compared to fresh catalyst. Leaching of active sites was also absent and the reaction proceeded via heterogeneous pathway.

**Scheme 5:**
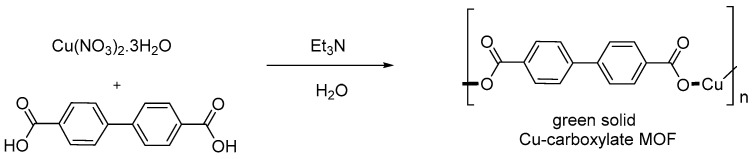
Preparation of Cu-carboxylate MOF.

**Figure 3:**
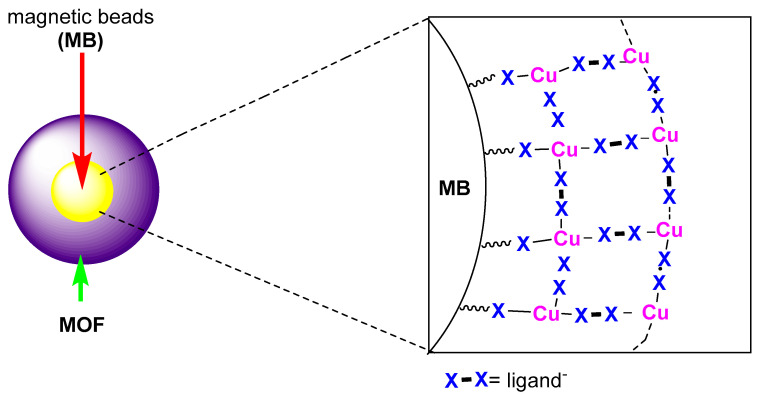
MOF-encapsulated MB catalyst.

Magnetic beads (MB) were encapsulated into catalytically active Cu-carboxylate MOF (Scheme 5) to obtain MOF-MB II (Figure 3), and its catalytic performance was evaluated in the Henry reaction [42]. Among the various conditions screened, the reaction between 2-nitrobenzaldehyde and nitromethane in the presence of MOF-MB II provided >99% yield in ethanol at room temperature after 23 h. Although a substrate scope with a wide range of aldehydes was studied with this catalyst, moderate to high yields are obtained in the range of 38–99% yield. The catalytic activity was retained for up to seven cycles, but the yield decreased in the eighth cycle. Further, ICP analysis indicated 0.4 μg/mL of Cu in each cycle. Characterization of the spent catalyst should have provided more insight into catalyst deactivation.

### 2.5. Sm Metal Nodes as Active Sites

A series of lanthanide MOFs [La(L1)_2_]_n_·1n(DMF)H·3n(DMF), [Ce(L1)_2_]_n_·1n(DMF)H·2n(DMF), [Sm(L1)_2_]_n_·1n(HCONH_2_)H·2n(HCONH_2_), [La(L2)(HL2)(H_2_O)(DMF)_2_]_n_ and [Ce(L2)(HL2)(H_2_O)(DMF)_2_]_n_ [L1: 2-acetamidoterephthalic acid and L2: 2-benzamidoterephthalic acid] were prepared by hydrothermal conditions [43]. Structural analysis of these MOFs indicates the existence of catalytically active sites that can behave as Lewis acids. Among the various catalytic conditions screened to achieve maximum efficiency of these solids for the reaction between benzaldehyde and nitromethane, [Sm(L1)_2_]_n_·1n(HCONH_2_)H·2n(HCONH_2_) exhibited a 70% yield with a TON value of 23.8 at 70 °C in water after 36 h. The substrate scope of [Sm(L1)_2_]_n_·1n(HCONH_2_)H·2n(HCONH_2_) was also surveyed, and the highest activity was obtained for the reaction of 4-nitrobenzaldehyde with nitromethane to give 98% yield of the desired product, with a TON value of 32.6 under identical conditions. The activity of this solid was retained for three cycles, with a minor decrease in 4th and 5th cycles. Although FT-IR and powder XRD data of the catalyst before and after the reaction remain identical, the cause of the slight catalyst deactivation should have been possible if the spent catalyst was characterized by relevant techniques. Hot filtration experiment showed the absence of Sm in the liquid reaction media.

## 3. MOFs as Organocatalysts for the Henry Reaction

### 3.1. MOFs with Primary Amine Sites

**Figure 4:**
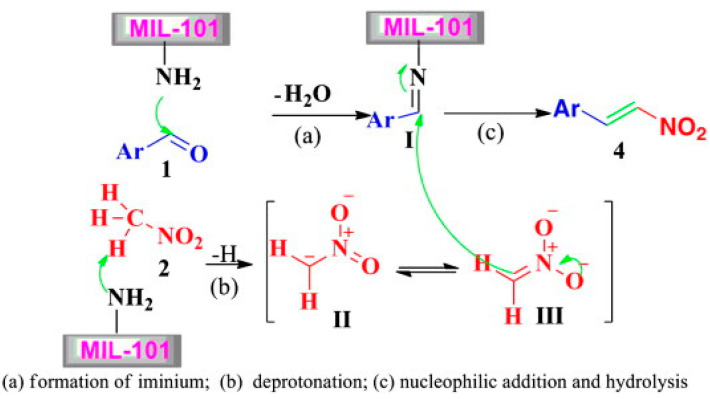
Proposed mechanism for the activation of benzaldehyde and nitromethane at the amino groups of MIL-101 metal-organic framework. Reprinted with permission from.44 Copyright (2012) Elsevier.

Ethylene diamine was incorporated into MIL-101(Cr), obtaining different degrees of well-dispersed, amino-functionalized, catalytically active, Cr-oxo secondary building units (SBUs) [44]. This solid was employed in the Henry reaction between nitromethane and benzaldehyde. An excellent conversion with moderate selectivity was obtained in polar protic solvents. On the contrary, poor conversion but high selectivity was achieved when using apolar solvents. Besides the solvent, the degree of amino functionalization was crucial to the catalytic performance of the MOF, since very high amine loading was detrimental, given the plugging of the pores, which disfavor the diffusion of the reactants into the cavities. The best performance with a TON of 9.4 and a TOF of 1.2 h^−1^ in toluene at 80 °C was achieved with a material containing 3.53 mmol_N_·g^−1^. The material was stable after the first run, since no leaching of ethylenediamine was detected by GC-FID. However, there was appreciable loss of ethylenediamine from the MOF in subsequent reuses (35% N leaching in the third run), which decreases the activity of the heterogeneous catalyst. Although the Cr-N bond between ethylenediamine and the SBUs was relatively labile, the Cr-O_2_CC_6_H_4_ SBU-linker bonds were very stable, since the MIL-101 structure was maintained after three catalytic cycles with only minor changes in the XRD patterns. It is worth mentioning that the synthesis of nitroalkenes was scalable up to 5 g (multigram scale) of the product without significant loss of yield. The proposed reaction mechanism involves the dual function of the anchored amino groups; on the one hand by the formation of imine intermediates with the benzaldehyde, and on the other hand by assisting the deprotonation of nitromethane. The addition of the resulting carbanion at the α-carbon of the nitromethane to the electrophilic carbon of the immobilized imine results in the C-C bond formation and desorption of the product from the MOF into toluene (Figure 4).

A novel procedure was reported for the synthesis of MIL-101(Cr)-NH_2_ through the reduction in MIL-101(Cr)-NO_2_, resulting in a highly porous material, possessing a BET surface area of 3262 m^2^·g^−1^, with respect to standard procedures (2525 m^2^·g^−1^) [45]. Such highly porous material is formed due to the low crystal size of the nitro-containing MOF precursor, which is maintained in the amino MOF (40–200 nm), much lower than that obtained with standard procedures (200–500 nm). The activity of the material was modest in the Henry reaction of benzaldehyde and nitromethane at 110 °C (TON of ca. 1) after 8 h under solvent-free conditions, but remarkable in line with the surface area of the material (higher for the more porous amino MOF, i.e., 95 vs. 79% benzaldehyde conversion). The higher activity of MIL-101(Cr)-NH_2_ is due to the larger amounts of exposed active basic sites in a highly porous nanocrystalline material, conferring superior activity.

The catalytic performance of copper/nickel azolate MOFs was modulated by the introduction of -NH_2_ or -OH groups to the aromatic ring of bispyrazolate linkers, which may interact with the benzaldehyde through hydrogen-bond interactions, promoting the Henry reaction with nitromethane in 2-butanol at 100 °C [46]. Thus, [Ni_8_(OH)_4_(H_2_O)_2_(1,4-bis(1H-pyrazol-4-yl)benzene)_6_] (NiBDP), [Ni_8_(OH)_4_(H_2_O)_2_(2-Hydroxo[1,4-bis(1H-pyrazol-4-yl)benzene])_6_] (NiBDP-OH) and [Ni_8_(OH)_4_(H_2_O)_2_(2-Amino[1,4-bis(1H-pyrazol-4-yl)benzene])_6_] (NiBDP-NH_2_), exhibited TON values (expressed in mol of nitrostyrene formed per mol of -H, -NH_2_ or -OH site) of 1.1, 1.3 and 2.0, respectively. Indeed, the presence of the -NH_2_/-OH groups increases the basicity of the solid, as measured by the pH of a MOF suspension in water. The incorporation of the -NH_2_ or -OH groups with high dispersion in the crystalline porous framework increased its activity with respect to the homogeneous linkers, which do not show activity with respect to the blank reaction, probably due to its self-deactivation (oligomerization) by H-bonding interactions. A hot-filtration test confirmed the heterogeneous nature of the MOF catalysts and XRD analysis of the spent materials showed the retainment of their structural integrity.

NH_2_-Tb-MOF was employed as a catalyst for the solvent-free reaction between benzaldehyde and nitromethane at 90 °C, exhibiting a TON of 6.7 after 24 h [47]. XPS analysis indicated that the Lewis basicity of the N atom in the amino group is maintained after the MOF formation, despite the conjugation effects at the Tb-O bonds. Thus, the authors considered that the exposed -NH_2_ groups in the MOF channels are believed to be the active sites of the material. However, although the similar conversion obtained with aniline or aminoterephthalic acid supports this hypothesis, no control reaction using the non-functionalized Tb-MOF was performed to rule out the activity of the Tb-oxo clusters. The structure and performance of NH_2_-Tb-MOF was maintained after five cycles, with some changes, especially at low diffraction angles, which were attributed to the adsorption of the reactants in the MOF.

To finalize this subsection, we will highlight that the introduction of the lysine amino acid was successfully achieved using the open Zr sites of MOF-808 as anchoring centers. Based on the elemental analysis and ^1^H-NMR spectrum of the digested MOF, there are two molecules of lysine coordinated by the carboxylic group to the Zr cluster (exchanged by the formate capping agent). This amino-acid-containing MOF resulted in an active amino-containing heterogeneous catalyst for the Henry reaction at room temperature under solvent-free conditions. The performance of the amino acid MOF is competitive with respect to amino-functionalized mesoporous organosilica, obtaining TOF values > 20 h^−1^ [48].

### 3.2. MOFs with Secondary Amine Sites

Post-synthetically modified MIL-101-NH_2_ with aziridine, in one step, results in covalently attached basic methyl amino groups at the organic linkers of the MOF [49]. The BET surface area of the starting MOF decreases from 2000 to 1200 m^2^·g^−1^ after the functionalization of 36% of the amino groups; thus, a large part of the area remains available for catalysis. In contrast to the large pore windows of MIL-101 (ca. 1.5 nm), other microporous MOFs (e.g., MOF-5, UiO-66, MIL-53) lose a large part of the porosity after the functionalization. Nevertheless, the UiO-66 solids containing secondary amines as active sites (28% functionalization) were able to catalyze the Henry reaction between benzaldehyde and nitromethane. The reaction was extremely fast, achieving a TON value close to 100 after just 2 h of reaction at room temperature in toluene. The high thermal stability of the catalyst ensures the structural integrity of the UiO-66 framework during the C–C-bond-forming reactions.

Free secondary amine groups present in a novel MOF linker containing well-defined pyridine and carboxylates as metal (i.e., Cu) anchoring points, were active catalysts for the Henry reaction [50]. The MOF consisted of a 2D polymeric (with a number of C–H···O interactions are present in the third dimension) architecture, with 7.59 × 7.00 Å^2^ open channels occupied by the non-coordinated water and DMF molecules, where the reaction between benzaldehyde and nitroethane to produce the nitro alcohol product in water, as a solvent, took place at 75 °C (with a TON of 42 after 40 h). Marginal yields were obtained when employing either the linker or metal salt alone, highlighting the importance of a rigid MOF structure to disperse the metal sites and amine groups for adequate catalytic performance. The mechanism proposed involves metal, Lewis basic sites and the ligand and/or water assistance to the nitroalkane deprotonation and C–C bond formation. The MOF retains its activity and structure after five reaction cycles, indicating the heterogeneous nature of the catalytic system.

A 3D-like 2D-layered Zn MOF composed of terphenyl dicarboxylate and DABCO ligands, coordinated to Zn by only one nitrogen atom, was employed as the Lewis base catalyst due to the presence of free nitrogen groups from the linker [51]. These open Lewis basic sites are located in an ordered manner inside the 1D microporous channels of the MOF, whose structure is maintained after the activation at 120 °C. A TON value of 48 was obtained for the neat Henry reaction between 4-nitrobenzaldehyde and nitromethane after 120 h at 60 °C. Both XRD and FTIR analyses of spent MOFs confirm the maintenance of the structure after four reaction cycles, without significant loss in either catalytic activity or Zn leached. Further analysis of the spent MOF indicates the exchange of the occluded DMF solvent molecules by the reaction substrates, which are adsorbed in the channels.

A final class of secondary amine containing MOFs are DUT-72 and DUT-90 materials, which consisted of copper coordinated to 1,3-phenylebis(azanetriyl)tetrabenzoate (mpbatb) and to DABCO by one of its N atoms, to accessible Cu sites pointing into the pores [52]. Both are excellent MOF catalysts for the Henry reaction between 4-nitrobenzaldehyde and nitromethane, with DUT-90 being more active than DUT-72. The Cu_4_(mpbatb)_2_(dabco)_1.45_ and Cu_4_(mpbatb)_2_(1,3-bib)_0.5_(dabco)_1.85_ materials that are still present open Cu sites with Lewis acidity. In DUT-72, the amount of potentially accessible Cu-sites is almost three times higher than in DUT-90 at comparable DABCO amounts. However, similar yields of the Henry adduct were obtained with both samples (up to 77%) at 50 °C after two days. Although the MOF structure withstands the reaction conditions after three cycles, the activity significantly decreases after the second cycle.

### 3.3. MOFs with Amide Sites

Zn-MOFs containing carboxylic linkers bearing free amido groups were active heterogeneous catalysts for the diastereoselective Henry reaction in methanol at 70 °C after or 48 h [53]. The zinc sites in the MOF were coordinated with both the free carboxylic acid sites of the acetamidoterephthalate linker, as well as to bipyridyl linker through the N donor atoms. The TON obtained was 32, with a TOF of 27 h^−1^. The MOF stability after two reaction cycles was confirmed by XRD and FTIR, which also showed no significant loss in activity. However, the authors do not propose the free amido group as a basic organocatalytic site through the H-bond activation of benzaldehyde. Instead, the only catalytic site considered in the proposed reaction mechanism was the Zn(II) at the SBUs. In another precedent, these MOFs were employed for the same reaction using water as the solvent, achieving TON values of 31 under the same conditions as in methanol, but in a sustainable and non-toxic aqueous medium [54]. Furthermore, the same authors highlighted the possible effect of basic environment provided by the amide backbone for increasing the overall yield of the reaction [55].

A different example of urea MOF is found in tripodal carboxylic acid linkers coordinated with copper ions, where the resulting network was stabilized through H-bonding with the assistance of amide groups [56]. Every Cu_4_O_4_ tetranuclear copper cluster is associated with four carboxylic ligands, four molecules of water and four μ_3_-methoxy ions. Two of the carboxylate groups of the ligand associate with the two neighboring tetranuclear clusters, while the carboxylic acid group remains uncoordinated and orients itself towards the open channels. The Cu-MOF was employed in the Henry reaction of nitroethane and benzaldehyde at 70 °C in methanol, obtaining a TON of 30 without losing activity or structural stability after four reaction cycles.

### 3.4. MOFs with Urea Sites

**Figure 5:**
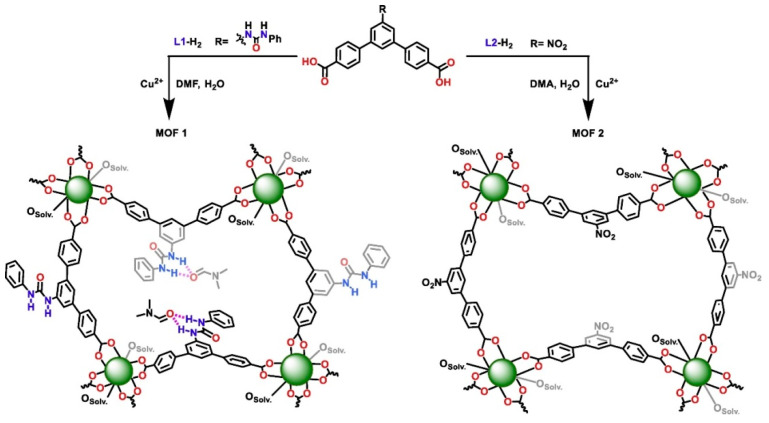
Synthesis of urea containing MOFs from V-shaped dicarboxylate linkers. Reprinted with permission from [57]. Copyright (2020) Elsevier.

Beside primary amino groups acting as basic (and covalent) catalysts, amide-type urea scaffolds acting as N–H acid (hydrogen bonding) sites were incorporated as linkers of MOFs [57]. The urea containing the V-shaped dicarboxylate linker reacted with Cu(II) to generate 2D MOFs (Figure 5) with catalytic activity for C–C-bond-forming reactions, such as the Henry reaction between benzaldehyde and nitromethane in methanol at 60 °C, resulting in a TON of 55 and a TOF of 2.3 h^−1^. In order to be active, the MOF needs to be treated with anhydrous methanol, CH_2_Cl_2_, and heated at 100 °C under vacuum for 2 h. This is due to the presence of hydrogen-bonded DMF guest molecules interacting strongly with the NH of the urea groups. Thus, prior to reaction, the occluded DMF must be removed from the pores of the MOF, since, otherwise, the TON obtained with the pristine MOF is only 6.7. A control reaction with the same copper MOF in the absence of the urea groups does not catalyze the Henry reaction (with a TON of 6). The advantages in terms of site-isolation of the urea moieties with respect to the homogeneous catalyst, which self-associates resulting in its deactivation, are clear when comparing their catalytic performance in the reaction between 4-nitrobenzaldehyde and nitromethane. The TON for the heterogeneous catalyst was 63, with respect to the 12 molecules of nitroalkene produced per urea site in the case of the homogeneous ligand. This is due to the isolated and unblocked urea sites in the MOF, which are ready for the activation of the carbonyl group of the aldehyde substrate. In contrast, the homogeneous ligand suffers its dimerization through intramolecular hydrogen bonding between two independent urea sites in two different molecules of the homogeneous catalyst, decreasing the number of free urea active sites.

A highly stable UiO-67 with urea moieties in the biphenyl linkers was prepared and activated in THF to remove the DMF interacting with the urea groups [58]. However, the catalytic activity of the pure urea Zr-MOF in the Henry reaction was poor (TON values of 3.6 in THF after 24 h at room temperature). The steric hindrance of the urea groups may block the access of the substrates to the inner active sites, thus utilizing only the surface-urea active sites. The incorporation of only 36 mol% of the urea moieties in the UiO-67 MOF allows for the maintenance of the porosity of the pure UiO-67 (pore diameters of 21.5 and 12 Å). The pure UiO-67-urea significantly decreases its pore diameters (12 and 9 Å) as well as the surface BET area with respect to the mixed urea-bpdc linkers (1550 vs. 390 m^2^·g^−1^). The mixed UiO-67-urea/bpdc outperforms the pure UiO-67-urea with a TON value of 14 after 24 h. This strategy allows the incorporation of bpdc and urea groups within the same crystal, as opposed to a physical mixture of bulk UiO-67 and UiO-67-urea, which does not improve the results obtained with the pure UiO-67-urea (TON value of 3.6). The homogeneous 1,3-diphenylurea catalyst displayed an intermediate behavior between the pure urea and mix-linker MOF, exhibiting a TON of 9. The immobilization of some urea moieties in the MOF scaffold enables spatial constraints that avoid the oligomerization-deactivation occurring with the homogeneous catalyst.

Urea containing UiO-68 Zr-MOF was prepared from two mixed terphenyldicarboxylate linkers, one of them with (trifluoromethyl)phenyl)ureido active sites anchored to one of the benzene rings, under microwave conditions [59]. The material has very large pores, which are needed to incorporate the sterically demanding phenylurea moiety inside the cavities, which becomes filled with the active sites. As a result, the surface area decreases from ca. 3500 to 2600 m^2^·g^−1^ in the case of the mixed linker MOF, and 300 m^2^·g^−1^ for the pure urea containing MOF. SEM images, combined with a mapping of the fluorine atoms from the urea containing linker, indicate the homogeneous distribution of these sites through the MOF crystal, without the phase segregation present in the case of physically mixed MOF phases. Liquid ^1^H-NMR analysis of the digested MOF indicates that the urea moiety is preserved during the MOF synthesis under mild conditions. As described in the case of UiO-67-urea, this UiO-68 isoreticular mixed-linker urea containing catalysts presents a better performance (TON of 18) of both the pure urea containing MOF (TON of 12) and the urea containing homogeneous ligand (TON of 8), in the Henry reaction of 4-methylbenzaldehyde with nitromethane in THF at room temperature. This is due to the higher porosity of the mixed-linker MOF and the stabilization of the urea groups in the rigid framework. The MOF can be recovered and reused without structural damage or loss of activity.

A summary of the catalytic performance of various MOFs in terms of TONs and the active sites in promoting the Henry reaction is summarized and shown in Table 1. It can be seen that the TON values oscillate between 2 and 100 molecules converted per active site for the reaction temperatures between room temperature and 100 °C (Table 1).

## 4. Conclusions and Summary

Different strategies for the incorporation of acid-base catalytic sites with activity in the Henry reaction, using MOFs as multifunctional platforms, are highlighted here. On the one hand, the incorporation of active sites into the MOF inorganic nodes resulted in a widely explored strategy to disperse transition metal catalysts in a controlled manner. Most of the studies report the successful recovery and recycling of the metallic active sites, without apparent loss in performance or framework crystallinity. However, the performance of such heterogeneous metal-containing catalysts in terms of activity, selectivity and stability should be compared under similar reaction conditions. The often-different solvents, temperatures and reaction times employed with the different systems make it difficult to establish structure–performance correlations. On the other hand, the incorporation of catalytically active organic functions inside the metal–organic architecture is a promising strategy to avoid the use of additional metals, which may be prone to leach from the framework. Therefore, we have commented on some recent works on the use of heterogeneous organocatalysis with MOFs for this particular Henry reaction. In this case, the design of the linkers is of paramount importance in order to strongly attach robust, but still active, organic functions as active sites.

Beside the MOF composition, the porous architecture and crystal size have a tremendous importance in terms of its catalytic performance. Thus, it will be important to take the textural properties of the designed MOF into account, to rule out any diffusion control that may interfere with the actual reaction taking place at the active site. In this sense, the use of computational techniques such as DFT or molecular dynamics could be a powerful tool to model the catalytic steps taking place during the Henry reaction and compare them with the experimental kinetic studies and in situ characterization. We are confident that more advanced modeling and characterization of MOF catalysts of C-C-bond-forming processes will be taken into account, thus enriching the chemistry of MOF-catalyzed organic processes. Finally, new chiral MOFs should be designed to achieve nitro aldol products with high ee values under mild reaction conditions. This is one of the fields that are currently less explored, and a higher number of studies is to be explored in the near future. This direction of research would certainly open new avenues for material, as well as organic, chemists.

## Figures and Tables

**Table 1:** Catalytic performance of the MOFs employed as solid catalysts in the Henry reaction between benzaldehyde and nitromethane.

MOF	Active Site	T (°C), Solvent	TON	Ref.
Co_2_(bdda)_1.5_(OAc)_1_·5H_2_O	Co	70, water	27.7	30
[Cd_2_(Cu(salen))(DMF)_3_]·DMF·3H_2_O	Cd/Cu	45, methanol	70	31
Cd_3_(CuL)_8_·(DMF)_6_·(H_2_O)_8_·(NMe_2_)_2_	Cd/Cu	60, methanol	86	32
{Cd_2_(Lglu)_2_(bpe)_3_(H_2_O)}·2H_2_O	Cd	RT, methanol ^a^	35.6	33
[Zn_3_(TCPB)_2_·2H_2_O]·2H_2_O·4DMF	Zn	70, neat ^b^	8	34
[Zn_2_(hfipbb)_2_(4-bpdh)].(DMF)_0.5_	Zn	60, methanol	32.3	35
[Cu_3_(pdtc)L_2_(H_2_O)_3_].2DMF.10H_2_O	Cu	70, neat	5.9	36
HKUST-1	Cu	100, toluene	5.6	37
NbO type-Cu MOF	Cu	50, neat	13.4	38
Cu-BTC	Cu	70, neat ^b^	4	39
HP-MOF	Cu	-	-	40
HKUST-1@MMS	Cu	75, water ^b^	18.2	41
MOF-MB II	Cu	RT, ethanol ^b^	19.8	42
[Sm(L1)_2_]_n_·1n(HCONH_2_)H·2n(HCONH_2_)	Sm	70, water	23.8	43
MIL-101-NH_2_ NiBDP-OH NH_2_-Tb-MOF	NH_2_	80, toluene	9.4	44
	OH	100, 2-butanol	2.0	46
	NH_2_	90, neat	6.7	47
MIL-101-NHR Cu-amide MOF	NHR	RT, toluene	100	49
	NHCOR	75, water	42	50
Zn-MOF-DABCO Zn-amide MOF Cu-urea MOF UiO-67-urea	NHR	60, neat	48	51
	NHCOR	70, methanol	32	53
	NHCONH	60, methanol	55	57
	NHCONH	RT, THF	14	58
UiO-68-urea	NHCONH	RT, THF	18	59

RT: room temperature. ^a^ nitroethane was employed instead of nitromethane. ^b^ p-nitrobenzaldehyde was employed instead of benzaldehyde.

## Data Availability

Not Applicable.
